# Characteristics of fatal insulin overdoses

**DOI:** 10.1007/s12024-022-00511-3

**Published:** 2022-08-09

**Authors:** Lilli Stephenson, Corinna van den Heuvel, Melissa Humphries, Roger W. Byard

**Affiliations:** 1 School of Biomedicine, The University of Adelaide, Adelaide, South Australia 5000 Australia; 2 School of Mathematical Sciences, The University of Adelaide, Adelaide, South Australia 5000 Australia; 3 Forensic Science SA (FSSA), Adelaide, South Australia 5000 Australia

**Keywords:** Coronial, Insulin, Diabetes, Suicide, Toxicology

## Abstract

This study was undertaken to review fatal cases of insulin overdose in South Australia (SA) over a 20-year period to assess rates and characteristics of insulin-related deaths among insulin-dependent diabetics and non-diabetics for all manners of death. Records from the National Coronial Information System (NCIS) and Forensic Science SA (FSSA) were searched for all cases of fatal insulin overdose in South Australia (SA) between 2000 and 2019. Collected variables included age, sex, cause of death, scene findings, manner of death, decedent medical and personal histories, biochemistry, toxicology, histopathology, and autopsy findings. Statistical analyses were performed using R (version 4.1.2). Forty cases of insulin overdose were identified in SA between 2000 and 2019. Twenty-nine cases (72.5%) were suicides, with the remaining cases classified as accidental or undetermined intent. Thirteen of the 22 insulin-dependent diabetics (59%) had a history of depression, 10 of whom had previously demonstrated suicidal ideation. The current study has shown that suicides using insulin among insulin-dependent diabetics are equally as prevalent, if not more so than fatal accidental insulin overdoses. This can largely be attributed to insulin-dependent diabetic access to a potentially lethal substance. Suicide prevention strategies should focus on insulin-dependent diabetics with a history of depression, particularly for those with access to rapid-acting insulin.

**Supplementary Information:**

The online version contains supplementary material available at 10.1007/s12024-022-00511-3.

## Introduction

Synthetic insulin has been an effective drug for the maintenance of normal blood glucose levels for type I and type II diabetics since its discovery and isolation in 1921 [1]. Excessive insulin administration may, however, cause profound hypoglycaemia, subsequent brain damage, and even death, if not correctly diagnosed and promptly treated. While non-fatal accidental insulin overdoses are relatively common among diabetics [2–5], fatal suicidal insulin overdoses are much less frequent. Such cases present significant difficulties for forensic toxicologists and pathologists in the death investigation process sometimes due to a lack of medical history at the time of autopsy and analytical limitations in the laboratory. While there are numerous case reports and reviews of fatal and non-fatal insulin overdoses including accidents [6–8], homicides [9–17], and suicides [18–66], there are few longitudinal retrospective reviews of fatal insulin overdoses from a single institution. Therefore, a study was undertaken to review fatal cases of insulin overdose in South Australia (SA) over a 20-year period, from 2000 to 2019 to assess rates and characteristics of insulin-related deaths among insulin-dependent diabetics and non-diabetics.

## Materials and methods

Records from the National Coronial Information System (NCIS) and Forensic Science SA (FSSA) were searched for all cases of insulin overdose in South Australia (SA) over a 20-year period (2000–2019). An initial comprehensive search was conducted in the NCIS for cases from 1 July 2000 to 31 December 2019 for all pharmaceutical substance-related deaths using the search terms “insulin” and “hypoglycaemia” which returned results for all cases where insulin or hypoglycaemia was found to cause or contribute to death. Only cases where the cause of death was recorded as “insulin toxicity” or “insulin overdose” (or other comparable terminology) were included in the current study. Cases where insulin administration or hypoglycaemia were recognized but not related to the cause of death were excluded. Relevant cases (*n* = 40) were then cross-referenced against autopsy and toxicology reports from FSSA to retrieve missing and additional information. Cases which occurred prior to 1 July 2000 were sourced from FSSA records only, as NCIS records prior to 1 July 2000 are unavailable. Collected variables included age, sex, cause of death, scene findings, manner of death, decedent medical and personal histories, biochemistry, toxicology, histopathology, and autopsy findings. Age was categorized into the following groups: young person (15–24 years), adult (25–64 years), and elderly (65 + years).

Routine toxicological analyses (for alcohol and common drugs) were conducted at FSSA. However, FSSA does not currently have a validated method for the analysis of insulin in post-mortem specimens. Therefore, post-mortem samples were sent to an external laboratory (SA Pathology, Royal Adelaide Hospital, North Terrace, Adelaide, South Australia) for quantitative analysis of insulin and C-peptide.

**Fig. 1:**
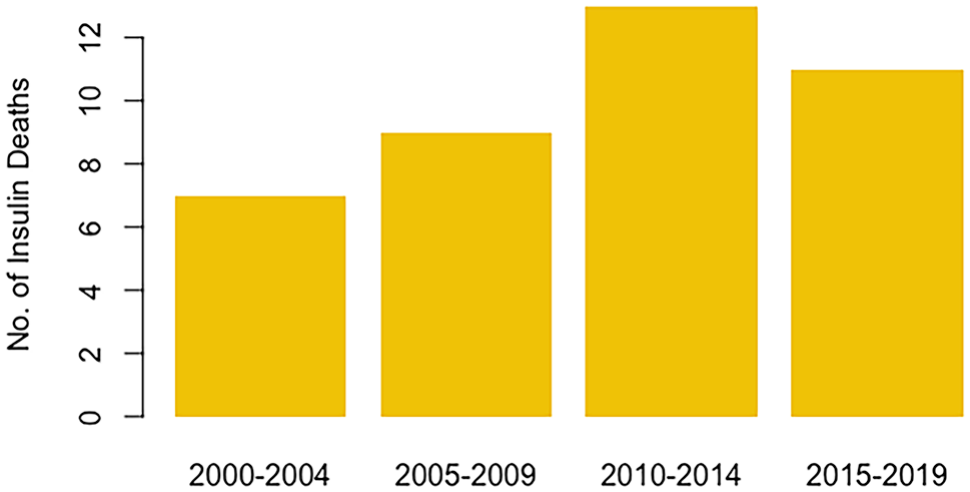
Number of insulin overdoses in South Australia between 2000 and 2019, divided into 5-year bins

Statistical analyses were performed using R (version 4.1.2). A Poisson regression was used to characterize trends over time. While statistical analysis was performed on the number of deaths in each year, the number of deaths is presented as 5-year bins in Fig. 1 to ensure individual cases within small cohorts are not identifiable. A Welsh two-sample *t*-test was used to determine significance in the age and sex distribution of the study population. Cohorts of fewer than five are reported as “less than five” within the text to ensure small cohorts are not identifiable.

Ethics approval for the data used in this study was granted by the University of Adelaide Human Research Ethics Committee (H-2020–033) and the Justice Human Research Ethics Committee (CF/21/2821).

## Results

### Overview

#### Rate of deaths

Forty cases of fatal insulin overdose were identified in SA between 2000 and 2019 with an average of two deaths per year. Poisson regression demonstrated no significant change in the rate of deaths over this period (*p* = 0.427); i.e., the rate of deaths per year across the 20-year study period was consistent (Fig. 1, see Appendix A for full statistical details).

#### Demographic profile

A total of 87.5% (*n* = 35) of decedents were aged 25–64 years; the remaining five decedents were either younger or older. The average age of all decedents was 49.5 years and included 19 females (mean 48 years) and 21 males (mean 51 years) with no significant difference in the age/sex distribution of the study population (*p* = 0.489).

#### Scene findings and external examination

Twenty-three deaths occurred at the home address of the decedent, nine in hospital, less than five at places of accommodation (hotel, motel etc.), and less than five on public property. Of those found on public property, all were found inside their motor vehicle. At the death scene, a suicide note was found in 19 cases. Evidence of insulin administration (syringes, needles, insulin vials, etc.) was documented in 26 cases (65%). While the type of insulin administered was not recorded in a large proportion of cases (61%), Novorapid, Actrapid, and Lantus were frequently encountered, either alone or in combination. External examination of the deceased revealed injection sites in 23 cases (57.5%), most frequently on the abdomen or thighs.

#### Histopathology

Of the 40 cases, a full autopsy with histopathological examination of tissue specimens was only performed in 31 cases. An autopsy was not performed in six cases as the cause of death was ascertained from hospital admission records in conjunction with a pathology review; histopathological examination of tissue specimens was not undertaken in two cases as the cause of death could be ascertained from the toxicology and biochemistry findings alone and severe putrefaction prevented accurate assessment of tissue morphology in the final case. Histopathology revealed evidence of lung oedema and congestion with or without the presence of early bronchopneumonia in 30 cases (97%). Neuropathology revealed evidence of hypoglycaemia-induced neuronal necrosis in 17 cases (54%), with normal neuronal architecture in the remaining 14 cases. For the cases with region-specific neuropathology findings, neuronal necrosis was preferentially observed in the cerebral cortex and hippocampus (*n* = 10), followed by the cerebellum (*n* = 6), basal ganglia (*n* = 5), and brainstem (*n* = 2).

#### Toxicology

Of the 40 cases where insulin overdose was implicated in the cause of death, 33 were attributed to insulin toxicity alone (82.5%) and the remaining seven cases were attributed to mixed drug toxicity. For cases where the cause of death was recorded as mixed drug toxicity, the drugs that were co-administered and considered contributory to the death were most often benzodiazepine sedatives (e.g., diazepam and alprazolam), antidepressants (e.g., amitriptyline and venlafaxine), and one case with co-administration of the semi-synthetic narcotic analgesic, hydromorphone. The full toxicology and biochemistry findings for the 7 cases of mixed drug toxicity are presented in Appendix B.

#### Biochemistry (SA pathology)

Post-mortem blood samples were submitted to an external laboratory for quantitative analysis of insulin and C-peptide. Insulin and C-peptide levels were reported either in full or in part for 31 cases (Table 1). The C-peptide level was reported without a corresponding insulin level in one case, and three cases only reported an insulin level. The range for insulin and C-peptide levels for these cases were 1–3200 mU/L (mean 594 mU/L) and 12–553 pmol/L (mean 131 pmol/L), respectively.

**Table 1:** Summary of case findings for all fatal insulin overdoses

Case no.	Cause of death	Scene findings	External autopsy findings	Histopathology	Insulin level (mU/L)	C-peptide level (pmol/L)	PMI (days)	Comment
1	Fresh water drowning associated with insulin toxicity	Nil	Nil	Lungs: oedema/congestion Kidneys: no diabetic glomerular/vascular changes Brain: normal neuronal architecture	1100	NA	1	-
2	Hypoglycaemia and asphyxia	Found with plastic bag over their head, IV line in arm with insulin	Nil	Lungs: oedema/congestion Kidneys: no diabetic glomerular/vascular changes Brain: normal neuronal architecture	83	NA	2	-
3	Bronchopneumonia complicating hypoglycaemic and anoxic/ischaemic brain damage due to insulin toxicity	Nil	Injection marks on lower abdomen	Lungs: oedema/congestion with early bronchopneumonia Kidneys: no diabetic glomerular/vascular changes Brain: widespread necrosis of cerebral cortex and basal ganglia	NA	NA	1	Hospital admission blood sample not available for testing
4	Acute bronchopneumonia due to insulin toxicity	Syringes, a large quantity of medication and several suicide notes	Nil	Lungs: oedema/congestion with acute bronchopneumonia Kidneys: markedly autolysed, focal areas of interstitial chronic inflammation/scarring Brain: normal neuronal architecture	6.7	NA	1	Insulin degraded during period of coma*
5	Insulin toxicity	Nil	Injection site on left lower thigh	Lungs: oedema/congestion Kidneys: diffuse cortical atrophy, diffuse diabetic glomerulosclerosis Brain: normal neuronal architecture	> 300	< 200	2	-
6	Consistent with insulin overdosage	Nil	Multiple injection sites on upper abdomen	Lungs: oedema/congestion Kidneys: no diabetic glomerular/vascular changes Brain: normal neuronal architecture	NA	NA	4	Blood specimen not suitable for analysis
7	Insulin toxicity	Used insulin syringes and suicide note	Multiple injection sites across upper abdomen and dorsal aspect of left hand	Lungs: oedema/congestion with focal early acute bronchopneumonia Kidneys: mildly autolytic, mild vascular sclerosis Brain: normal neuronal architecture	3100	< 200	3	-
8	Oxazepam and insulin toxicity	Nil	Injection site on lower abdomen	Lungs: oedema/congestion Kidneys: marked vascular sclerosis, diffuse/nodular glomerulosclerosis Brain: normal neuronal architecture	2200	< 200	3	-
9	Insulin toxicity	Nil	Multiple injection sites on upper abdomen	Lungs: oedema/congestion with focal early bronchopneumonia Kidneys: mild diabetic microvascular and glomerular changes Brain: possible early red cell change in Purkinje cells	1600	< 200	3	-
10	Insulin toxicity	Nil	Autopsy not performed	Autopsy not performed	NA	NA	NA	Hospital admission blood sample not available for testing
11	Insulin toxicity	Empty medication packets, empty insulin syringes and a suicide note	Seven injection sites on abdomen	Lungs: oedema/congestion with focal early bronchopneumonia Kidneys: mild glomerulomegaly with diffuse/focal nodular glomerulosclerosis Brain: microscopic focus of axonal and neuronal loss in basal ganglia and evidence of neuronal injury in cortex and cerebellum	190	< 200	4	-
12	Insulin toxicity	Suicide notes and several empty vials of insulin	Injection site on left side of abdomen	Lungs: oedema/congestion Kidneys: enlarged glomeruli Brain: normal neuronal architecture	15	< 100	4	-
13	Attributed to insulin overdose	Nil	Multiple injection sites on abdomen	Lungs: oedema/congestion Kidneys: scattered sclerotic glomeruli, patchy lymphocytic infiltrate Brain: normal neuronal architecture	2.6	100	4	Gross haemolysis of blood specimen, results unreliable*
14	Hypoxic brain injury due to benzodiazepine and insulin toxicity	Several empty medication packets and an insulin syringe	Nil	Lungs: oedema/congestion Kidneys: no diabetic glomerular/vascular changes Brain: severe and diffuse anoxic encephalopathy of cerebellum	NA	553	5	Insulin level not quantitated
15	Insulin toxicity	A suicide note and several empty insulin syringes	Multiple injection sites on lower abdomen and both thighs	Lungs: oedema/congestion Kidneys: focal nodular glomerulosclerosis Brain: normal neuronal architecture	160	< 166	2	-
16	Presumed insulin toxicity	Found with empty insulin syringes	Marked putrefaction and decomposition	Marked putrefaction and decomposition prevented accurate assessment of tissue morphology	NA	NA	11	Marked putrefaction inhibited analysis of a post-mortem blood specimen
17	Toxic effects of insulin	Suicide note	Injection site in right antecubital fossa	Lungs: oedema/congestion Kidneys: no diabetic glomerular/vascular changes Brain: prominent focal red degeneration-type change of nerve cells in hippocampus	410	< 100	4	-
18	Insulin toxicity	Suicide note	Autopsy not performed	Autopsy not performed	NA	NA	NA	Hospital admission blood sample not available for testing
19	Attributed to insulin toxicity	19 empty vials of Humalog insulin and syringes	Nil	Lungs: early haemorrhagic acute pneumonia Kidneys: no diabetic glomerular/vascular changes Brain: normal neuronal architecture	2.5	< 100	5	Extended PMI*
20	Insulin toxicity	Empty Actrapid insulin vial and syringe		Lungs: oedema/congestion with early bronchopneumonia Kidneys: no diabetic glomerular/vascular changes Brain: prominent focal red degeneration-type change of nerve cells in hippocampus and cortex	260	< 100	2	-
21	Hypoxic-ischaemic encephalopathy and toxic effects of insulin	Empty insulin vials in bin	Injection mark on lateral thigh	Lungs: oedema/congestion Kidneys: no diabetic glomerular/vascular changes Brain: extensive ischaemic necrosis of cortex, cerebellum, pons, midbrain and medulla	350	113	4	-
22	Insulin toxicity	Suicide note	Injection site on abdomen	Lungs: oedema/congestion Kidneys: consistent with diabetic change Brain: normal neuronal architecture	290	< 100	4	-
23	Insulin and temazepam toxicity complicated by aspiration pneumonia	Suicide notes, a will, empty insulin syringes and an empty packet of temazepam	Multiple injection sites over lower abdomen	Lungs: oedema/congestion, patchy aspiration pneumonia Kidneys: no diabetic glomerular/vascular changes Brain: possible early neuronal red cell changes	29	< 100	3	Early putrefaction, gross haemolysis of blood sample*
24	Insulin and venlafaxine toxicity	5 empty insulin vials and empty medications packets in kitchen bin	Injection site on left lower abdomen	Lungs: oedema/congestion with early bronchopneumonia Kidneys: consistent with diabetic change Brain: early neuronal red cell change in hippocampus	920	< 100	2	-
25	Mixed drug toxicity (insulin and hydromorphone)	Numerous empty medication packets, insulin vials and a suicide note	Multiple injection sites on lower abdomen	Histological examination was not undertaken since the cause of death could be determined from the toxicological and biochemical findings	93	< 100	3	-
26	Toxic effects of insulin	Several insulin syringes and a suicide note		Histological examination was not undertaken since the cause of death could be determined from the toxicological and biochemical findings	120	< 100	2	-
27	Insulin toxicity	Diabetic testing kits, insulin vials and a suicide note	Autopsy not performed	Autopsy not performed	NA	NA	NA	Hospital admission blood sample not available for testing
28	Insulin and alprazolam toxicity	Insulin vials and syringes, will and suicide note	Nil	Lungs: oedema/congestion Kidneys: mild intimal hyperplasia Brain: extensive red degeneration of nerve cells of hippocampus, cerebellum and cerebral cortex	1700	0	4	-
29	Toxic effects of insulin	Lantus and NovoRapid insulin pens	Nil	Lungs: oedema/congestion Kidneys: no diabetic glomerular/vascular changes Brain: red degenerative change of neuronal cells in hippocampus and cortex	3200	< 100	2	-
30	Hypoxic, ischaemic and hypoglycaemic encephalopathy due to insulin overdose	Suicide note	Multiple injection sites on abdomen and both thighs	Lungs: oedema/congestion Kidneys: chronic interstitial nephritis, focal sclerosis Brain: neuronal necrosis in hippocampus, basal ganglia and parietal cortex	310	< 100	8	Extended PMI*
31	Insulin toxicity	Insulin vials	Autopsy not performed	Autopsy not performed	NA	NA	NA	Hospital admission blood sample not available for testing
32	Insulin toxicity/overdosage	Suicide note, Lantus and NovoRapid insulin pens		Lungs: oedema/congestion Kidneys: sclerotic glomeruli Brain: early neuronal dark cell change within hippocampus	26	< 100	3	-
33	Consistent with insulin toxicity	Syringe and empty Lantus and Actrapid insulin vials	Injection site on right abdomen	Lungs: oedema/congestion Kidneys: no diabetic glomerular/vascular changes Brain: evidence of neuronal necrosis in hippocampus and basal ganglia	6	< 100	3	-
34	Attributed to insulin toxicity	15 empty Humalog insulin pens, Last Will and Testament nearby	Seven injection sites on lower abdomen	Lungs: oedema/congestion, focal acute pneumonia Kidneys: no diabetic glomerular/vascular changes Brain: potential dark-cell change in dentate nuclei, Purkinje cells, cortex and basal ganglia	22	< 100	3	-
35	Insulin toxicity	One empty Lantus and seven empty NovoRapid insulin pens	Several injection sites on right abdomen	Lungs: oedema/congestion Kidneys: consistent with diabetes Brain: normal neuronal architecture	1300	< 100	4	-
36	Hypoglycaemic brain injury due to insulin toxicity complicated by aspiration pneumonia	Suicide note and 8 empty NovoRapid FlexPen’s	Several injection sites on lower abdomen and upper thighs	Lungs: oedema/congestion with patchy early aspiration pneumonia Kidneys: consistent with diabetes Brain: early neuronal necrosis in hippocampus and superficial cortical neurons	1.9	< 100	4	Rapid acting (3–5 h) insulin administered, rapidly degraded*
37	Insulin toxicity	Numerous Novorapid insulin vials and an empty syringe	Injection site on lower right abdomen	Lungs: nil Kidneys: consistent with diabetes Brain: neuronal necrosis in cerebellum, dentate nucleus, cerebral cortex and hippocampi	< 1	47	4	Insulin degraded during period of coma*
38	Insulin toxicity	Suicide note detailing insulin overdose	Autopsy not performed	Autopsy not performed	NA	NA	NA	Hospital admission blood sample not available for testing
39	Hypoxic ischaemic encephalopathy following insulin and benzodiazepine overdose	Empty insulin syringe, suicide note	Autopsy not performed	Autopsy not performed	NA	NA	NA	Hospital admission blood sample not available for testing
40	Attributed to insulin toxicity	15 empty Lantus insulin pens, 2 empty NovoRapid insulin pens and a suicide note	Several injection sites on abdomen	Lungs: oedema/congestion Kidneys: consistent with diabetes Brain: red cell change in frontal cortex and temporal cortices and cerebellum	12.8	< 12	7	Extended PMI*

*NA* not available

^*^Insulin/C-peptide results unreliable

Insulin and C-peptide analyses were not performed in the remaining nine cases due to the unavailability of a hospital blood sample for analysis in seven cases and two cases where the blood sample was deemed unsuitable for analysis.

Identification and interpretation of insulin and C-peptide levels are dependent on the integrity of the post-mortem blood sample. Insulin levels may be artificially lowered due to a prolonged post-mortem interval (PMI) which is associated with haemolysis of post-mortem blood samples and subsequent insulin degradation. The PMI ranged from 1 to 11 days across all 40 cases (mean = 3.6 days). Of the 31 cases where insulin and C-peptide levels were reported, the results in five cases were considered to be unreliable due to a significant PMI (i.e., 5 days or longer) or evident haemolysis of the blood sample. Furthermore, three cases demonstrated a prolonged period of survival (coma) between insulin administration and death allowing time for insulin to be degraded, or the administration of rapid-acting insulin which is also rapidly degraded. Therefore, the insulin and C-peptide levels reported in the eight cases highlighted above were significantly lower than would be reflective of a death due to insulin toxicity.

Average (mean) insulin and C-peptide levels were calculated for the remaining 23 cases where the biochemistry results were considered accurate and reliable. The average blood level of insulin and C-peptide for these cases was 816 mU/L and 144 pmol/L, respectively, with an approximate ratio of 6:1. The C-peptide level was frequently denoted as “ < 100 pmol/L”; therefore, this is a significant underestimate of the real ratio. For reference, a normal fasting insulin level is between 0 and 12 mU/L and a normal C-peptide level is between 300 and 1600 pmol/L.

#### Decedent histories

The medical history of decedents revealed a history of depression in 23 cases (57.5%), 12 of whom had previously demonstrated suicidal ideation. However, fewer than five decedents did not have a recorded history of depression but had previously demonstrated suicidal ideation according to the personal histories available. Proximal risk factors for intentional self-harm noted in decedent histories included family/relationship issues in seven cases, significant health issues in five cases, and financial/employment/legal issues in three cases. More than half of decedents were insulin-dependent diabetics (*n* = 22, 55%) and five were not. The diabetic status of the remaining decedents was unknown. Thirteen of the 22 insulin-dependent diabetics (59%) had a history of depression, 10 of whom had previously demonstrated suicidal ideation.

#### Manner of death

The manner of death was suicide in 29 cases (72.5%) with the remaining 11 cases being of accidental or undetermined intent (27.5%).

### Accidental/undetermined deaths

#### Rate of deaths

Eleven cases of accidental or undetermined intent were identified with an average rate of 0.58 deaths per year. Poisson regression demonstrated no significant change in the rate of deaths over this period (*p* = 0.379).

#### Demographic profile

The average age of all decedents was 46.4 years and included 5 females (mean 48 years) and 6 males (mean 45 years) with no significant difference in the age/sex distribution of the cohort (*p* = 0.733).

#### Decedent histories

Decedent histories revealed a history of depression in seven cases, five of whom had previously demonstrated suicidal ideation. Financial/employment/legal issues were noted in one case. Six of the decedents were insulin-dependent diabetics (55%); the diabetes status of the remaining decedents was unknown.

### Suicide

#### Rate of deaths

Twenty-nine cases of suicide were identified with an average rate of 1.38 deaths per year. Poisson regression demonstrated no significant change in the rate of deaths over this period (*p* = 0.120).

#### Demographic profile

The average age of all decedents was 50.8 years and included 14 females (mean 48 years) and 15 males (mean 53.3 years) with no significant difference in the age/sex distribution of the cohort (*p* = 0.294).

#### Decedent histories

Decedent histories revealed a history of depression in 16 cases (55.2%), seven of whom had previously demonstrated suicidal ideation. Previous suicidal ideation was noted in less than five cases where there was no recorded history of depression. Family/relationship issues were noted in seven cases, significant health issues in five cases and financial/employment/legal issues in two cases. A suicide note was found in 19 cases (65.5%). Sixteen decedents were insulin-dependent diabetics (55.2%), three were not, and the diabetes status of the remaining decedents was unknown. Of those that were not insulin-dependent diabetics, the insulin was sourced either from their workplace or a family member.

## Discussion

Since its discovery and isolation, insulin has been an effective drug for the maintenance of normal blood glucose levels for type I and type II diabetics. However, self-administration dosing errors have been identified as a frequent cause of hospital admission among insulin-dependent diabetics, particularly among the young [2–5]. Excessive insulin administration can cause profound hypoglycaemia, subsequent brain damage, and even death if not correctly diagnosed and promptly treated. There are an abundance of case reports and literature reviews on fatal and non-fatal insulin overdoses involving accidents [6–8], suicides [18–66], and homicides [9–17]. In comparison to other compounds, insulin has, however, been considered a somewhat ineffective lethal agent due to the long period of time required to produce permanent damage and the ease with which an overdose may be diagnosed and treated [67–69]. Unfortunately, there is a lack of longitudinal reviews in the literature to assess long-term trends in fatal insulin overdoses for all manners of death.

Case reports and literature reviews of fatal insulin overdoses have identified likely underreporting due to laboratory and pathology limitations. Suspicion may not be aroused without a comprehensive medical or personal history, which is often not available at the time of autopsy. Cases may also be incorrectly classified as accidents due to an undiagnosed history of depression [8]. The literature has identified an association between diabetes and an increased prevalence of psychiatric disorders such as depression or suicidality, particularly in those with a long history of diabetes and those on insulin therapy [70–72]. At a poisons unit in Germany, the incidence of intentional insulin overdoses was significantly higher (85.3%) than accidental cases compared to other anti-diabetic compounds [73]. More than half (59%) of insulin-dependent diabetics in the current study had a medical history of depression, the majority of which had demonstrated suicidal ideation (77%). However, if the decedent has not been prescribed insulin or is not known to be an insulin-dependent diabetic, as was highlighted in this study, examination for evidence of exogenous insulin administration may not be considered. Even if examination is performed, puncture wounds may not be evident as the needle used to administer subcutaneous insulin is very fine which may further obscure cases [74]. At autopsy, evidence of subcutaneous insulin injection was only observed in 57.5% of cases in the current study. Case reports of insulin-related deaths often report the presence of insulin administration equipment at the death scene. In the current study, evidence of insulin administration (i.e., used syringes, insulin vials) was documented in just over half (65%) of cases. There also remains the possibility that such material may be removed or hidden by the decedent or by family/friends. Therefore, a presumptive cause of death of insulin toxicity should be based on a multitude of factors including the decedent’s medical history integrated with scene, autopsy, toxicology, biochemistry, and histology findings.

While cases of fatal insulin overdose are thought to be a rare occurrence, the current study suggests that while in low numbers, they continue to occur consistently over time. Furthermore, suicidal insulin overdoses are just as frequent, if not more so than accidental overdoses or those of undetermined intent. While significant sex/gender disparities are often observed in suicidal deaths with a male predominance [75], no differences were shown in age or sex distribution of the current study population. For insulin-dependent diabetics, an undiagnosed history of depression with or without suicidal ideation is particularly problematic as those on insulin therapy have access to a potentially lethal pharmacological suicide agent. As several decedents within this cohort had previously attempted suicide using insulin, it is important that appropriate medical monitoring is directed towards repeat-attempters with access to insulin [71]. In the current study, those who were not insulin-dependent diabetics and utilized insulin as a pharmacological suicide agent were either health professionals or had a relative/partner who had been prescribed insulin. However, for several of the cases deemed to be of accidental or undetermined intent, it is unclear how the decedent gained access to insulin.

In addition to difficulties in ascertaining manner of death, there are also limitations in the detection and confirmation of exogenous insulin administration which may contribute to the underreporting of cases. As noted, there are significant analytical limitations associated with post-mortem insulin and C-peptide detection and quantification. Insulin is known to be unstable in post-mortem blood samples due to the activity of insulin-degrading enzyme which is concentrated within red blood cells and released with haemolysis [76–78]. Traditional methodologies utilize radioimmunoassay and enzyme-linked immunoassays which are associated with limitations in identification, differentiation, and quantification [64]. For traditional immunoassays, insulin analogues have also been shown to be cross-reactive which has been a major obstacle in developing an immunoassay to effectively identify analogues or metabolites, with significant variation between assays [79–81]. Newer methods using liquid chromatography with tandem mass spectrometry (LC–MS-MS) have been developed in recent years with success in both quantification of insulin and differentiation between analogues [5, 64, 65, 67]. However, it is yet to be seen how accessible these new methodologies will be in terms of availability and cost.

For cases where reliable insulin and C-peptide levels were obtained, these were significantly outside the normal ranges. Some authors also consider the ratio of insulin to C-peptide (I:C) to be a useful measure to distinguish between exogenous and biological hypoglycaemia as C-peptide is only formed with release of endogenous insulin, and should not be > 1 in a healthy individual [82]. The approximate ratio of insulin to C-peptide was shown to far exceed the normal, healthy level in all decedents where reliable biochemistry results were available. However, the ratio of insulin to C-peptide has also been shown to change significantly in the post-mortem interval, even within 24 h [11]. If post-mortem blood is to be used, samples should be taken within 48 h of death (if possible) and from a peripheral site to limit the effects of post-mortem redistribution [11, 83]. Several other biological sample sites have been evaluated, with vitreous humor found to be the most promising as it is less susceptible to diffusion and the effects of decomposition, and thus is more reliable for determination of insulin and C-peptide [67].

At autopsy, pathological changes indicative of insulin overdose and hypoglycaemia are often nonspecific although hypoglycaemic neuronal necrosis may be observed [84–87]. Neuropathological animal studies show that blood glucose must persist at a level below 1 mM for at least 30 min for neuronal death to occur which has been confirmed by human autopsy studies [84–88]. While normal neuronal architecture was observed in 35% of cases, this could be indicative of a short survival period post-insulin administration, and thus, the presence or absence of neuronal pathology cannot be considered diagnostic of fatal hypoglycaemia without a detailed timeline of events. For decedents in the current study where extensive neuronal death was observed, this was generally correlated with a prolonged period of survival between insulin administration and death. Previous studies show that hypoglycaemic neuronal death occurs sequentially by brain region, first evident in the cerebral cortex and hippocampus, followed by the basal ganglia and thalamus as was shown in the present report [9, 87, 89].

This study has shown a consistent although low rate of insulin-related deaths over a two-decade period in a single Australian state. Further investigations are required to determine whether this trend is observed in other states and countries. Problems with pathological assessments and toxicological/biochemical evaluations may indicate, however, that the true number of these deaths may be higher and that some of the deaths considered to be accidental or undetermined may in fact be occult suicides. Therefore, suspicion and confirmation of insulin toxicity as the cause of death should be based on a multitude of factors including the decedent’s medical history integrated with scene, autopsy, toxicology, biochemistry, and histology findings.

## Key points

1. A low, yet consistent number of fatal insulin overdoses occurred in South Australia between 2000 and 2019
2. Suicidal insulin overdoses were significantly more frequent than accidental insulin overdoses, with more than two-thirds of cases found to be suicides
3. A large proportion of insulin-dependent diabetics who died due to fatal insulin overdose had a history of depression, often with previous suicidal ideation
4. There are significant analytical limitations associated with quantification of insulin and C-peptide in post-mortem samples
5. A finding of insulin toxicity as the cause of death should be based on a multitude of factors including the decedent’s medical history integrated with scene, autopsy, toxicology, biochemistry, and histology findings

## Supplementary Information

Below is the link to the electronic supplementary material.

Supplementary file1 (DOCX 13 KB)

Supplementary file2 (DOCX 18 KB)
