# Supplementary material for: Characteristics of fatal insulin overdoses
Source: Forensic Sci Med Pathol. 2022 Aug 9;18(4):429–41. doi: 10.1007/s12024-022-00511-3 (PMC9636090; doi:10.1007/s12024-022-00511-3)
Supplement: Supplementary file 1 — Supplementary file1 (DOCX 13 KB) [file 12024_2022_511_MOESM1_ESM.docx]

**APPENDIX A:** Poisson regression R output

##

## Call:

## glm(formula = Count ~ Year, family = "poisson", data = yeardat)

##

## Deviance Residuals:

## Min 1Q Median 3Q Max

## -2.00300 -0.79475 0.00984 0.38838 2.23285

##

## Coefficients:

## Estimate Std. Error z value Pr(>|z|)

## (Intercept) -43.27089 55.38574 -0.781 0.435

## Year 0.02187 0.02755 0.794 0.427

##

## (Dispersion parameter for poisson family taken to be 1)

##

## Null deviance: 21.868 on 19 degrees of freedom

## Residual deviance: 21.235 on 18 degrees of freedom

## AIC: 71.468

##

## Number of Fisher Scoring iterations: 5
