# Supplementary material for: Characteristics of fatal insulin overdoses
Source: Forensic Sci Med Pathol. 2022 Aug 9;18(4):429–41. doi: 10.1007/s12024-022-00511-3 (PMC9636090; doi:10.1007/s12024-022-00511-3)
Supplement: Supplementary file 2 — Supplementary file2 (DOCX 18 KB) [file 12024_2022_511_MOESM2_ESM.docx]

**APPENDIX B:** Toxicology and biochemistry findings for mixed drug toxicity cases

| Case no. | Drug | Specimen | Level* | Interpretation |
| --- | --- | --- | --- | --- |
| 1 | Carbamazepine | Blood | 8 | Non-toxic/therapeutic |
|  | Metoclopramide | Blood | 0.06 | Non-toxic/therapeutic |
|  | Oxazepam | Blood | 4.8 | Potentially lethal |
|  | C-peptide | Blood | <200pmol/L | Abnormally low |
|  | Insulin | Blood | 2200mU/L | Markedly elevated |
| 2 | Diazepam | Blood | 0.08 | Non-toxic/therapeutic |
|  | Nordiazepam | Blood | 0.1 | Non-toxic/therapeutic |
|  | Oxazepam | Blood | 0.2 | Non-toxic/therapeutic |
|  | Temazepam | Blood | 0.5 | Non-toxic/therapeutic |
|  | C-peptide | Blood | 553pmol/L | Within normal limits |
|  | Insulin | Blood | NQ | Present |
| 3 | Codeine | Blood | 0.02 | Non-toxic/therapeutic |
|  | Fluoxetine | Blood | 0.1 | Non-toxic/therapeutic |
|  | Temazepam | Blood | 0.2 | Non-toxic/therapeutic |
|  | C-peptide | Blood | Not detected | - |
|  | Insulin | Blood | 29mU/L | Mildly elevated |
| 4 | Diazepam | Blood | 0.7 | Non-toxic/therapeutic |
|  | Haloperidol | Blood | 0.03 | Non-toxic/therapeutic |
|  | Nordiazepam | Blood | 0.3 | Non-toxic/therapeutic |
|  | O-desmethylvenlafaxine | Blood | 11 | Potentially lethal |
|  | O-desmethylvenlafaxine | Liver | 14mg/kg | Potentially lethal |
|  | Oxazepam | Blood | 0.1 | Non-toxic/therapeutic |
|  | Quetiapine | Blood | 0.6 | Non-toxic/therapeutic |
|  | Quetiapine | Liver | 3.6mg/kg | Non-toxic/therapeutic |
|  | THC | Blood | 3ug/L | Non-toxic/therapeutic |
|  | Temazepam | Blood | 0.1 | Non-toxic/therapeutic |
|  | Venlafaxine | Blood | 17 | Potentially lethal |
|  | Venlafaxine | Liver | 34 mg/kg | Potentially lethal |
|  | Warfarin | Blood | 0.4 | Non-toxic/therapeutic |
|  | C-peptide | Blood | <100pmol/L | Abnormally low |
|  | Insulin | Blood | 920mU/L | Markedly elevated |
| 5 | Amlodipine | Blood | 0.6 | Non-toxic/therapeutic |
|  | Hydromorphone | Blood | 0.3 | Potentially lethal |
|  | Indomethacin | Blood | NQ | Present |
|  | Mirtazapine | Blood | 0.1 | Non-toxic/therapeutic |
|  | C-peptide | Blood | <100pmol/L | Abnormally low |
|  | Insulin | Blood | 93mU/L | Elevated |
| 6 | 7-aminonitrazepam | Blood | 0.52 | Potentially toxic |
|  | Alprazolam | Blood | 0.43 | Potentially toxic |
|  | Mirtazapine | Blood | 0.1 | Non-toxic/therapeutic |
|  | Nitrazepam | Blood | 0.04 | Potentially toxic |
|  | Temazepam | Blood | 0.2 | Non-toxic/therapeutic |
|  | C-peptide | Blood | Not detected | - |
|  | Insulin | Blood | 1700mU/L | Markedly elevated |
| 7 | Benzoyl ecgonine | Blood | 0.34 | Non-toxic/therapeutic |
|  | Oxazepam | Blood | 0.21 | Potentially toxic |
|  | Temazepam | Blood | 4 | Potentially toxic |
|  | C-peptide | Blood | NQ | Present |
|  | Insulin | Blood | NQ | Present |
| *mg/L unless stated otherwise NQ = not quantitated | | | | |
